# Procurement and early deployment of artificial intelligence tools for chest diagnostics in NHS services in England: a rapid, mixed method evaluation

**DOI:** 10.1016/j.eclinm.2025.103481

**Published:** 2025-09-11

**Authors:** Angus I.G. Ramsay, Nadia Crellin, Rachel Lawrence, Holly Walton, Stuti Bagri, Emma Dodsworth, Holly Elphinstone, Fergus Gleeson, Amanda Halliday, Kevin Herbert, Joanne Lloyd, Efthalia Massou, Raj Mehta, Stephen Morris, Pei Li Ng, Tracy O’Regan, Chris Sherlaw-Johnson, Naomi J. Fulop

**Affiliations:** a Institute of Epidemiology and Health Care, University College London, UK; b Research and Policy, Nuffield Trust, London, UK; c Department of Oncology, University of Oxford, Oxford, UK; d Public Contributor, Cambridgeshire, UK; e Department of Public Health and Primary Care, University of Cambridge, UK; f Public Contributor, Devon, UK; g Public Contributor, London, UK; h The Society and College of Radiographers, London, UK

**Keywords:** Artificial intelligence, Chest diagnostics, Radiology, Procurement, Implementation, Health services research

## Abstract

**Background:**

Artificial Intelligence (AI) may support accurate, efficient radiology diagnostics. However, little is known about implementing AI in clinical settings. In 2023, NHS England launched a programme funding 12 networks of 66 NHS Trusts to implement AI for chest diagnostics, including lung cancer.

**Methods:**

Rapid evaluation (March–September 2024) of procurement and early deployment of AI for chest diagnostics at network (n = 10) and Trust (n = 6) levels. We interviewed network teams, Trust staff, and AI suppliers (n = 51); observed planning, governance, and training (n = 57); and analysed relevant documents (n = 166). The NASSS framework guided thematic analysis.

**Findings:**

Procurement and deployment of AI took longer than anticipated. Procurement involved engaging selection panels, assessing tenders, and contracting AI suppliers. Preparation for deployment involved AI integration; governance processes; staff engagement and training; planning patient engagement; and collating impact data; patient communication plans varied and were still developing. Challenges included: engaging staff with high clinical workloads; staff’s limited AI knowledge, time to participate, and concerns over appropriate tool usage; managing unsuccessful suppliers’ responses; and varied local governance processes, IT systems, and data availability and quality. Enablers included: programme leadership’s support; networks sharing expertise and capacity; committed clinical, technical, and procurement specialists and AI suppliers; clinical champions; and dedicated project management.

**Interpretation:**

Implementing AI involved complex social and technical processes, requiring significant resources. Future implementation may benefit from ensuring sufficient time and capacity, ongoing stakeholder engagement at multiple levels, and greater consideration of patients and equity, diversity, and inclusion. Influential factors identified here mirror research on other healthcare innovations, suggesting AI may not address service challenges as straightforwardly as policymakers anticipate.

**Funding:**

10.13039/501100000272National Institute for Health and Care Research (NIHR), 10.13039/501100022224Health and Social Care Delivery Research programme (NIHR156380). NJF and AIGR are supported by NIHR Central London Patient Safety Research Collaboration. NJF is an NIHR Senior Investigator.

**Research in context**

**Evidence before this study**

AI may support accuracy and efficiency of radiology diagnostics services, but there is little published evidence on the procurement, deployment, and implementation of AI tools at scale in real-world clinical settings.

**Added value of this study**

Our study is one of the first to analyse real-world implementation of AI in radiology diagnostics at scale. Our analysis demonstrates that, in line with many healthcare innovations, procurement and preparation for deployment of AI tools are complex social and technical processes, requiring significant time and resources. We identify the key tasks involved and factors that influenced these processes, including the wider context, local capacity and infrastructure, and approaches to leadership and management of change.

**Implications of all the available evidence**

Real-world implementation of AI tools may be supported by ensuring sufficient time and capacity, ongoing stakeholder engagement at multiple levels, and greater consideration of patients and equity, diversity, and inclusion. While the factors we identify parallel other healthcare innovations, the relative novelty of AI—at policy, regulation, and service levels—meant there was limited guidance and knowledge on procurement and preparation for deployment: this increased uncertainty throughout these processes; further, the influential factors we identify suggest that AI may not address service challenges as straightforwardly as policymakers anticipate.

## Introduction

In the UK, health services face increasing pressures due to increasing population need, changes in clinical practice, and limited service capacity.^1^^,^^2^ There has been growing interest in the potential for artificial intelligence (AI) to support radiology services, e.g., by removing the need for some clinical interactions and improving diagnostic efficiencies.^2^, ^3^, ^4^, ^5^, ^6^, ^7^, ^8^, ^9^, ^10^, ^11^, ^12^

Evidence suggests AI might benefit diagnostic services by supporting decision-making, improving detection accuracy, reducing errors, increasing efficiency, and easing workforce burdens.^13^, ^14^, ^15^ However, little is known about real-world implementation, including procurement, preparation for deployment, experiences of staff, patients, and carers, and impact on effectiveness and costs—due in part to very few studies having been conducted on real-world implementation of AI tools for radiology diagnostics.^16^ As healthcare systems increasingly adopt AI tools, and given system leaders’ anticipation of transformative change,^1^^,^^17^ there is a pressing need for evidence to support implementation and use of AI, programme design, policy, regulation, and evaluation.

In July 2023, the Artificial Intelligence Diagnostic Fund (AIDF) was launched to support AI deployment for chest diagnostics, including lung cancer, across 12 imaging networks (bodies created to support innovation in and standardised use of imaging diagnostics across National Health Service (NHS) services across their local regions)^7^ and 66 of the 124 acute NHS Trusts (NHS organisations that provide a range of healthcare, e.g., acute hospital care or mental health care, to their local communities) in England.^18^ AIDF, which was led by NHS England, provided £21 m to services and aimed to increase diagnostic efficiency, reduce backlogs and waiting times, and ease workforce pressure. Improving chest diagnostics and lung cancer detection are national priorities due to high rates of late-stage lung cancer diagnosis,^3^ and efficient chest diagnostics may be central to managing any future respiratory disease pandemic.^19^

To help increase understanding of real-world implementation of AI tools in a high priority healthcare setting, we analysed procurement and preparation for deployment of AI tools as part of the AIDF programme. Our analysis was guided by the Non-adoption, abandonment, scale-up, spread, sustainability (NASSS) framework, which considers the social and technical factors that interact to shape planning, implementation, and uptake of technological innovations.^20^^,^^21^

Our analysis addressed the following questions:

1. How were AI tools for chest diagnostics procured and deployed as part of AIDF?
2. Which factors (e.g., context, technology, implementation processes, capacity to implement, and stakeholder characteristics) influenced procurement and early deployment of AI?
3. What are the lessons for future real-world implementation and evaluation of AI in diagnostics?

## Methods

This analysis was part of a rapid study (conducted March–September 2024, the first phase of a larger project, which will run to February 2026),^22^ guided by the NASSS framework.^20^ We conducted a combination of ‘light touch’ and ‘in depth’ case studies. For light touch cases (n = 7), data were collected at network-level only; in ‘in-depth’ cases (n = 3), data were collected at both network and Trust levels (two Trusts per network, i.e., a total of n = 6 Trusts across three networks). Using a purposive sample, we interviewed network teams, Trust staff, and AI suppliers (n = 51 interviewees). Given the rapid nature of the work, in each organisation we targeted a small number of interviewees who had direct experience of procurement and preparation for deployment of AI tools. As this study sampled only staff, rather than patients, carers, or the public, we did not sample interviewees in relation to ethnicity or socioeconomic status. We also conducted non-participant observations of planning, governance, and training activities (n = 57); and we reviewed relevant documents, including AI specifications and implementation plans (n = 166) (Table 1).

**Table 1:** Overview of data collected.

Site	Site type	Interviews	Observations	Documents
1	In-depth	6, of which: • Network: 2 • Trust A: 1 • Trust B: 3 Not interviewed: 1^a^	19 • Network: 5 • Trust A: 6 • Trust B: 8	19
3	In-depth	11, of which: • Network: 3 (includes 1 f/u^b^) • Trust A: 3 • Trust B: 5 Not interviewed: 1	19 • Network: 15 • Trust A: 2 • Trust B: 2	22
9	In-depth	8, of which: • Network: 3 (includes 1 f/u) • Trust A: 4 • Trust B: 1	7 • Network: 7	29
2	Light touch	2	0	16
4	Light touch	2	1	2
5	Light touch	5 (includes 1 f/u) Not interviewed: 1	2	12
6	Light touch	4^c^	6	15
7	Light touch	3^c^	0	6
8	Light touch	5 (includes 2 f/u)	2	19
10	Light touch	2	1	26
	Supplier 1	1	0	0
	Supplier 2	1	0	0
	Supplier 3	1^d^	0	0
**Total**		**51**	**57**	**166**

a 'not interviewed’ = staff who were approached, but did not respond to invitation for interview.

b 'f/u’ = ‘follow-up interview.

c Includes joint interviews.

d Supplier that was contracted by network that did not participate in this study.

Interviews were conducted by researchers with qualitative (AIGR, NC, RL, HW), quantitative (CSJ, SB, ED), and health economic (KH, EM) expertise, using topic guides structured around our research questions and the NASSS framework (Supplementary file A).^20^ Potential interviewees were approached by e-mail and provided with information and consent form, outlining the purpose of the work. Interviews were conducted only with informed consent (written or verbal); they took place online via videocall, and were audio-recorded and professionally transcribed. Interviews lasted between 45 and 70 min. To support rapid formative feedback, key points were summarised using Rapid Assessment Procedures (RAP) sheets structured around our research questions and guided by NASSS (Supplementary file B).^20^ Following this rapid analysis, we revisited full transcripts for coding. Full analysis focused on procurement and preparation for deployment of AI for chest diagnostics. Data were managed in Microsoft Excel. Coding was led by the qualitative researchers (AIGR, NC, RL, HW): key themes were identified around our research questions, then developed iteratively from the data; the analysis focused on the role of NASSS constructs. Formative findings were shared with participating networks throughout the study at national meetings and through a webinar at the end of the project (while many networks were still in the process of deploying AI tools): responses from attendees provided validation of our findings and recommendations for how future research might evaluate implementation and impact of AI tools.

### Ethics

Ethical approval was obtained from the University College London Ethics Committee (27037/001).

### Role of funding source

This study was funded by National Institute for Health and Care Research (NIHR) Health and Social Care Delivery Research programme (NIHR156380). The funders did not have a role in study design, data collection and analysis, writing of the manuscript, or the decision to publish. The views and opinions expressed are those of the authors and do not necessarily reflect those of the NIHR or the Department of Health and Social Care.

## Results

This section presents the data collected for this analysis (Table 1) and a timeline of key events related to procurement and preparation for deployment of AI tools (RQ1). We then discuss factors influencing these processes (RQ2), presenting local examples and overarching themes linked to NASSS constructs. Finally, we identify lessons for future implementation and evaluation, including data requirements (RQ3).

We recruited 10 of the 12 networks participating in AIDF: of the two that did not participate, one had joined the programme late, while the other was facing extensive challenges in implementation; neither felt they had the capacity to participate in the work. Three of the 54 people approached for interview did not take part: this was as a result of individuals not responding to invitations rather than refusal to participate. Staff interviewees included radiologists and radiographers of varied seniority, IT and digital diagnostics managers, procurement specialists, and project managers. The majority of activities observed were governance meetings (including national meetings between network leads, local planning and oversight meetings); we also observed a small number of other activities, including a training session and a local review of a patient safety risk assessment. We were not refused access to any activities we sought to observe. Documents analysed included specifications and scoring summaries for all procurement processes, local implementation plans, data protection impact assessments, and patient safety risk assessments.

### Timeline for procurement and preparation for deployment

**Fig. 1:**
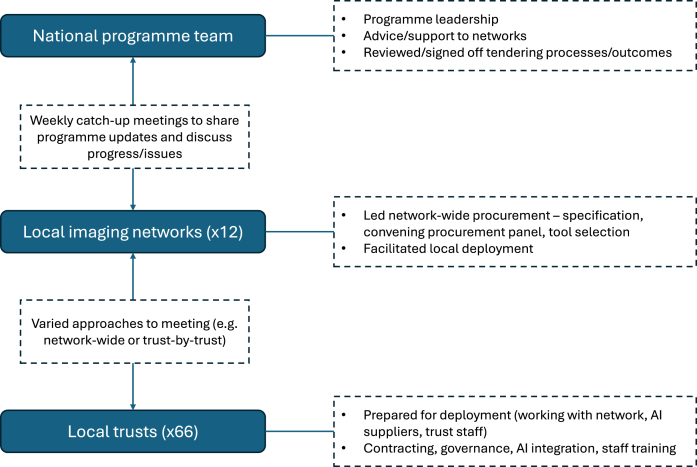
**Key groups and activities.** Note. ‘AI’ = ‘Artificial Intelligence’.

Fig. 1 outlines key groups involved in AI procurement and deployment. Contracts were originally expected to be agreed by November 2023 but delayed substantially: in the in-depth networks, contracts were signed in March 2024, May 2024, and September 2024, respectively. Deployment in clinical services was expected to start in December 2023, but in practice began in some sites in May 2024. In the in-depth networks, AI went ‘live’ in clinical practice over the periods September–December 2024, July–December 2024, and January 2025, respectively. AI tools were operational in clinical services in 24 of 66 Trusts by November 2024 and in 43 Trusts at time of writing (June 2025).

### Factors influencing procurement and preparation for deployment

**Fig. 2:**
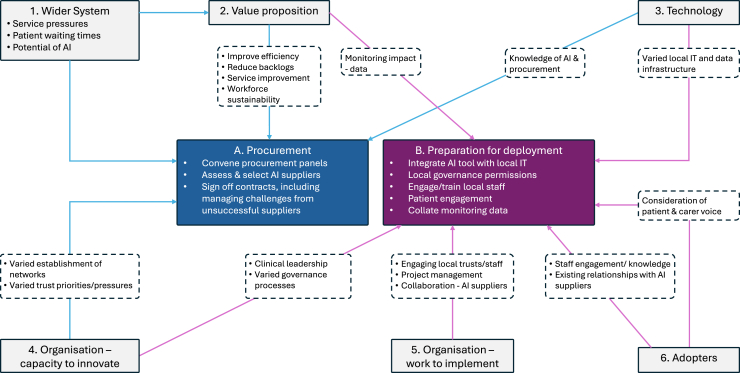
**Factors influencing procurement and preparation for deployment of AI, organised by NASSS framework.**^20^ Note. ‘AI’ = ‘Artificial Intelligence’; ‘IT’ = ‘Information Technology’.

Below, we present factors influencing procurement and preparation for deployment of AI tools for chest diagnostics. Throughout, we present overarching themes, citing examples from different networks and Trusts to illustrate the range of experiences and approaches (e.g., model of implementation, project management). Fig. 2 summarises the overall analysis, and illustrative quotes are presented in Supplementary file C.

#### Policy/service context

The NASSS construct of the ‘wider system’—i.e., policy and service context—influenced significantly both procurement and preparation for deployment (Fig. 2, box 1) (quote: Supplementary file C, section 1). There were longstanding policy-level concerns about growing pressure on services and patient waiting times, and increasing recognition of potential contributions AI might make to address these challenges.

Reflecting the NASSS construct of ‘value proposition’ (Fig. 2, box 2), these priorities shaped AIDF’s short-term aims to increase diagnostic efficiency and reduce backlogs, and longer-term aims to improve diagnostic services and waiting times, reducing workforce burden.

Service pressures shaped initial rapid timelines. While capacity and service pressures drove the introduction of AI, limited capacity among Trust staff–already stretched during the busy winter period–also slowed procurement and deployment progress.

#### Procurement: processes and influential factors

Local imaging networks worked with participating Trusts on key tasks, including convening procurement panels, tender selection, and signing contracts (Fig. 2, box A). Network-level procurement meant that networks could potentially choose different AI tools, but that all Trusts within a network would have to use the tool selected by their network.

### Convening local panels to procure AI

Panels of local representatives were convened to develop AI tool specifications and assess AI suppliers’ tenders (quotes: Supplementary file C, section 2a). Reflecting the NASSS construct of ‘knowledge required to use the technology’ (Fig. 2, box 3), procuring AI demanded diverse expertise, spanning AI, clinical pathways, local diagnostics information systems, and procurement processes. Therefore, engaging these panel members was a critical task.

Networks varied in their maturity and relationships with local services (reflecting the NASSS construct of ‘capacity to innovate’ (Fig. 2, box 4)). More established networks were better placed to identify and recruit appropriate procurement panel members.

There was also variation in AI expertise at network-level: some leads had prior experience, while others were new to AI procurement. We observed several examples of networks sharing learning at national meetings, including obstacles and potential solutions (e.g., sharing specification documents). Procurement panels included diverse expertise, including: clinical specialists (e.g., radiologists, radiographers, and chest clinicians), technical experts (e.g., digital and imaging systems, information governance), while a range of other expertise was covered more variably (e.g., finance, procurement) (data: Supplementary file D). However, some panel members reported lacking confidence in their AI knowledge and ability to differentiate effectively between AI tenders.

### Assessing and selecting tenders

Procurement panels assessed tenders against specification criteria, covering e.g., clinical effectiveness, cost, and environmental and social impact (quotes: Supplementary file C, section 2b). This activity reflected NASSS constructs of ‘work needed to implement change’ and ‘knowledge required to use the technology’ (Fig. 2, boxes 3 and 4). Specifications were developed with support from the wider programme (e.g., national team sharing specification templates, and more experienced network representatives sharing example procurement questions).

All potential AI suppliers had first to be approved for the national procurement framework for this programme (demonstrating compliance with requirements of e.g., Medicines and Healthcare products Regulatory Agency, GDPR, and Cyber Essentials certification). Suppliers chose the networks to which they submitted a tender. Networks received a mean of 6.6 tenders (range 4–9 tenders) (data; Supplementary file E).

Panel members assessed tenders independently, before reaching consensus on scoring and selecting the preferred tender. However, several reported that information provided did not facilitate informed decision-making, e.g., due to large amounts of supporting documentation submitted, resulting in some members having to deal with too much information, some of which was seen as excessively technical; this potentially increased the likelihood of key details being missed. Some networks mitigated this by requiring panel members to focus solely on criteria reflecting their area of expertise. Panel members reported little tailoring of tenders to local settings (e.g., AI tool integration schematics did not reflect local IT infrastructure as set out in specifications). Demonstrations of the tool working on local imaging IT systems, were identified as a potential aid to decision-making.

Selection criteria covered a range of features, including service quality, costs, and equity, diversity, and inclusion. The majority of selected tools scored highest on quality. While equity was a required consideration in all procurement processes, interviewees indicated that this did not generally change the selection made, and some perceived this element as ‘box-ticking’.

Some procurements also faced challenges where participating Trusts had established relationships with AI suppliers, and clinicians preferred to work with a known quantity. Independent scoring against specification criteria was important in ensuring selection of a tool that reflected the network overall.

### Contracting

Following National Programme leadership approval, local contracting processes commenced (quotes: Supplementary file C, section 2c). These were slowed by issues raised by unsuccessful AI suppliers. All networks received queries from suppliers, including e.g., requests for feedback to strengthen future applications, disagreements with decisions (some suggesting bias), and legal challenges. Several Network representatives found this a novel experience, potentially resulting from AI being more commercialised than settings with which they were familiar. The challenges were discussed frequently at weekly national network meetings, where the national team offered guidance, but noted the need for networks and Trusts to work with their procurement and legal teams to manage these challenges.

While procurement was coordinated at network level, contracting varied due to the autonomy of NHS organisations and a history of independent procurement and contracting across Trusts. Although imaging networks could work across these organisations, they lacked the authority to sign contracts on their behalf. Consequently, some networks designated a ‘lead Trust’ to manage contracting, supported by a memorandum of understanding that other Trusts would adopt this contract. In other networks, each organisation completed its own contracting. These processes delayed preparation for deployment by several weeks.

### AI tools selected

**Fig. 3:**
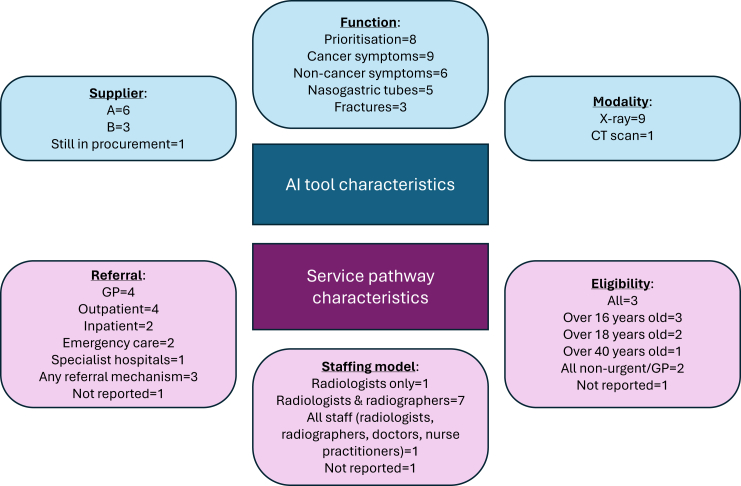
**AI tool and service pathway characteristics.** Note. Numbers refer to number of networks; for several categories, networks covered more than one characteristic (e.g., functions, referral methods); therefore, in several cases, numbers exceed 10. ‘AI’ = ‘Artificial Intelligence’; GP = ‘General Practitioner’.

In the networks studied here, only two of the sixteen AI suppliers that tendered were selected (Supplementary file E). The AI tools selected varied in terms of diagnostic modality (e.g., focusing on X-ray or CT scan), and functions (Fig. 3). Key functions included prioritisation of urgent cases for review and identification of potential symptoms. AI tools also worked differently within service pathways, in terms of referral route (e.g., GP referrals, inpatient referrals, or emergency care referrals), eligibility for inclusion (e.g., variations in age groups covered), and the staff accessing AI readouts (e.g., radiologists only, radiologists and radiographers only, or all clinical staff). Importantly, all decisions still had to be made by humans: AI readouts were only to be examined once human readers had conducted their assessments.

#### Preparation for deployment: processes and influential factors

Preparing for deployment occurred at Trust-level, supported by local networks, and included integrating AI, governance processes, staff engagement and training, planning patient engagement, and collating impact data (Fig. 2, box b).

### Integrating AI with local IT systems

AI tools had to be integrated with local IT systems, such as Radiology Information Systems (RIS) and Picture Archiving Communications Systems (PACS), reflecting the NASSS construct, ‘technology’ (Fig. 2, box 3) (quotes: Supplementary file C, section 3a). Due to variations in RIS and PACS suppliers and systems across Trusts, integration often had to be repeated in each Trust, rather than as a single network-level process.

One network had already standardised IT systems across Trusts, avoiding such duplication. This enabled network-level development and network-wide implementation of codes to prioritise patient scans on radiology worklists (i.e., lists of scans for review by radiologists). Another network used the AIDF programme to align their local IT infrastructure to a common PACS platform: although this would potentially facilitate future AI implementation, the complexity and scale of this process delayed deployment of AI tools.

Changes to workflow involved the prioritisation of scans for reporting (e.g., one network categorised prioritisation using ‘critical’, ‘high’, and ‘standard’ labels, with critical to be reported first) and the AI read being visible for staff to review after completing their report. One network also implemented standardised x-ray reporting codes which enabled timely and appropriate onward referrals, e.g., a code for high suspicion of cancer, requiring urgent follow-up CT within 72 h.

Project management was key to integration tasks. We observed project meetings attended regularly by Trust and AI supplier staff, working to agreed timelines, and operating a risk register where integration tasks were a central focus. Close engagement of clinical leads (e.g., network and Trust leads, often experienced radiologists), local IT teams (e.g., PACS managers), and AI suppliers (including staff with expertise in relevant platforms and processes, such as secondary capture) was evident across sites. Members were responsible for specific tasks, e.g., developing IT capabilities, testing the AI tool using retrospective imaging data, implementing a period of ‘shadow mode’ before the AI was live in clinical practice, and updating standard operating procedures. This was especially important where the AI suppliers had no experience of integrating AI with NHS systems.

### Obtaining local governance permissions

Obtaining information and patient safety governance approvals was crucial (quotes: Supplementary file C, section 3b). Variations in local systems (reflecting the NASSS construct of ‘capacity to innovate’ (Fig. 2, box 4)) made network-wide progress challenging. Trusts operated independent governance procedures, with differing meeting structures and documentation templates, making many unwilling or unable to process forms that did not comply with their local requirements. Consequently, a single approach could not be applied across all Trusts: this increased workload required to obtain local governance permissions.

Governance meetings occurred infrequently in some cases, resulting in delays while awaiting official sign-off. Conversely, we observed instances of local governance processes being convened to facilitate more timely sign-off. For instance, a Trust Clinical Safety Officer joined an extended project meeting to assess hazard identification (HAZID) documents, enabling faster approval.

Addressing these issues involved substantial collaboration between local Trust and supplier staff. Network- and Trust-level planning meetings were frequently attended by supplier team members, including AI experts and relationship managers. Several networks (n = 6) noted the importance of clinical champions (with clinical and AI expertise) to facilitate navigation of local processes.

The use (or otherwise) of dedicated project managers was an important enabler of governance processes. Where present, there was greater capacity to facilitate progress (e.g., arranging meetings, managing timelines, engaging governance contacts) and local knowledge of relevant individuals. Where absent, these tasks were covered by local healthcare staff (in addition to their daily responsibilities, with implications for all activities) or network representatives (with limited capacity to perform this function across multiple Trusts). Interviewees suggested that dedicated project management made an important difference to progress of the work.

### Engaging and training staff

It was important that staff knew how to use the tool and understood its limitations, i.e., AI should only act as decision support, reflecting the NASSS constructs of ‘work needed to implement change’, ‘staff adopters’, and ‘knowledge needed to use technology’ (Fig. 2, boxes 3, 5, and 6) (quotes: Supplementary file C, section 3c). A common approach was to identify ‘super users’—clinicians who would be trained, and then roll out training to their colleagues (n = 8).

Observed super user training sessions were led by the AI supplier team: the AI tool was presented on the local diagnostic system and key features were demonstrated and discussed, e.g., highlighting areas of interest in scans, a bar indicating AI confidence in its interpretation, and how to report clinician agreement with AI interpretation. The sequence and nature of processes was also emphasised, i.e., the requirement for human readers to complete their assessment before checking the AI report, and that the human reader’s interpretation should take precedence over AI.

One site’s hazard assessment raised questions about how the organisation ensured new and established staff were trained before using the AI tool. This was an issue because diagnostics system access was not set at an individual level. The decision was to maintain a formal training log for diagnostics staff; however, it was recognised that such a solution would be much harder to manage safely if AI were to be used in emergency departments, where staff numbers accessing the diagnostic system was larger and less consistent.

In some networks, staff reported mixed responses to the prospect of AI, with concerns raised by more senior clinicians about the potential impact of AI making decisions without clinician input, and where accountability lay in the event of a condition being missed. However, training did not address these issues: early and ongoing engagement may have helped address these concerns.

### Considering patient and carer engagement

Reflecting the NASSS construct of ‘patient and carer adopters’ (Fig. 2, box 6), we explored the extent to which services had considered engaging patients and carers on the issue of AI processing their scans (quotes: Supplementary file C, section 3d). As interviews occurred pre-deployment, many sites and networks were still to finalise their approach, however we found variation in their plans. Some networks intended to share information through posters, leaflets, and local/social media (n = 4); one network considered communicating directly with patients (though the mechanism was uncertain); two networks had no plans to inform patients; while another would share information only upon patient request. Meetings highlighted the potential to frame AI implementation as a “good news story” for the NHS and patients, whilst also recognising the importance of transparency, particularly as patients could learn of AI use independently by accessing their records. However, this sat alongside the fact that more generally, there is no formal requirement for innovations of this kind to be disclosed i.e., as the tools were classified as a medical device there was no change in how data were used, and it was stipulated that tools should not influence clinical decisions.^23^^,^^24^

### Providing data to evaluate impact of AI

The AIDF programme required that participating networks and Trusts provide “benefits metrics” data assessing the impact of AI, reflecting the NASSS construct of ‘value proposition’ (Fig. 2, box 2) (quotes: Supplementary file C, section 3e). Data were to be shared at baseline (pre-deployment) and at 6-monthly timepoints post-deployment into clinical practice (i.e., as clinical decision support and prioritisation of cases). Sharing baseline data was a condition for funds being released, demonstrating the programme’s prioritisation of monitoring and evaluation.

In practice, many networks experienced challenges in providing these data rapidly (n = 7) noting the complexity of data reporting requirements. Several measures requested relied on accessing and linking data from more than one source, and the data infrastructure in many Trusts was insufficient to process such access and linkage automatically. Consequently, many sites had to dedicate additional resources to accessing and processing these data. The process of providing the data was facilitated by centralised data systems managed at a network level and support from a data analyst and/or manager where available.

## Discussion

System-level recognition of pressures on healthcare services and political and managerial optimism about AI’s potential to alleviate these are increasing. However, little published evidence on real-world implementation and impact of AI on radiology diagnostics exists: this introduces risk into national and international deployment initiatives. In this first-of-its-kind study, our analysis addresses some of these gaps by analysing real-world procurement and preparation for deployment of AI for chest diagnostics, including lung cancer, as part of a major programme covering much of the NHS in England, taking a theory-driven approach to identify influential factors. The lessons we identify may be of value to national and regional health service leaders, as AI is anticipated to be increasingly central to health services,^12^ and chest diagnostics may be key to managing any future respiratory disease pandemic.^19^

A recent review notes substantial gaps in real-world evaluation of implementation and use of AI for diagnostics in clinical settings, for example, it found no studies on procurement.^16^ This study contributes evidence on procurement and preparation to deploy AI tools, demonstrating that they are complex socio-technical processes. Network-level procurement of AI tools involved considerable engagement with local organisations to identify selection panel members and organise AI selection processes. These activities required much more time and resource than anticipated and available, placing demands on clinical, technical, and managerial staff. Preparation for deployment demonstrated substantial variations in Trust-level radiology IT systems, governance processes, and data infrastructure. We found benefits in factors such as using dedicated project management. These findings underscore the value of engaging stakeholders (e.g., national leadership, network teams, Trust clinical, managerial, and technical staff, and AI suppliers) throughout the process, reflecting previous published research on implementing AI in radiology services.^25^^,^^26^

Many factors identified in our analysis—including context (at system, organization, and service levels), leadership and engagement approaches, technological considerations, use of data, and local capacity and resourcing (e.g., finance, people)–reflect established findings on implementation of other innovations, digital or otherwise,^21^^,^^27^, ^28^, ^29^, ^30^ and NASSS framework constructs.^20^ A common issue was the novelty of AI, suggesting a need for greater understanding of and guidance and education on AI and its implementation at system (policy and regulation) and organizational levels. The use of AI tools as decision support, rather than operating autonomously, aligned with existing UK regulation and public attitudes.^16^

Our analysis addresses some key evidence gaps around real-world implementation of AI in radiology diagnostics at scale. We provide insights on factors influencing procurement and preparation for deployment across diverse networks of healthcare organisations in the English NHS. Our hybrid sample let us understand how networks approached procurement and deployment, providing in-depth insights on how preparation for deployment progressed at Trust-level. Our theory-driven approach let us consider these processes from diverse perspectives and reflect on how different factors interacted to shape implementation progress. Our rapid approach enabled timely formative feedback of learning to services and programme leadership.

Our analysis has several limitations. First, due to implementation delays, we could not study several important issues, including day-to-day use of AI in clinical practice, impact on care, outcomes, and costs, and adaptation and sustainability of AI tools over time. Second, due to the tight timeline of this phase of our evaluation, we were unable to interview several important groups, including patients and carers who experience diagnostics supported by AI, and clinical and managerial staff who work in or oversee care pathways affected by chest diagnostics. Third, this evaluation was of a large, funded pilot conducted in NHS services in England (covering 66/124 acute NHS trusts in England): therefore our lessons may apply less to other NHS organisations that did not take part in the AIDF programme, other healthcare systems with different regulatory arrangements, or to situations where services are commissioning AI tools without financial incentives to do so. Fourth, while interviewees were closely engaged in aspects of procurement and preparation for deployment of the AI tools, we recruited small numbers of people within networks and NHS trusts: therefore, we may have missed a more diverse range of perspectives, e.g., from other team members who had less positive views of the programme. However, staff feedback at our webinar provided some validation of our findings.

Further research is needed to explore real-world implementation, use, and impact of AI in clinical settings, ideally across different health and regulatory contexts. Multi-site, mixed-method studies, including perspectives from patients and carers and clinical, managerial, and technical healthcare staff would offer important insights, e.g., addressing the ethical question of when and how patients should be informed about AI use. Our findings demonstrate variations in how AI use was to be communicated to patients, with many plans still in development. Our recent review also noted the lack of evidence focused on patient views and/or experiences of AI-supported care, suggesting that future implementation should include the voices of those impacted by AI advancements,^16^ Therefore, future research should include patient-focused topics, such as the impact of AI use on empathy and human connection, patient experiences of AI-supported care and ethical debates about transparency, consent, and safety.^16^ Longer-term research on the impact and sustainability of such services may enable full analysis of the relationship between early diagnostics, healthcare interventions, long-term health outcomes, and cost-effectiveness. Research designs should also reflect the dynamic nature of AI innovations, e.g., using rapid cycles of data collection and formative feedback to support responsive service development. In the event that anticipated changes in regulatory frameworks^12^ result in extended functions of AI in diagnostics (e.g., greater autonomy of AI reporting), routine monitoring and independent study would be important sources of assurance and learning.

Procurement, deployment, and evaluation of the impact of AI are complex social and technical processes, requiring significant resources (e.g., time, capacity, expertise). Future implementation may be strengthened by planning sufficient time and/or capacity to support implementation; ongoing stakeholder engagement at multiple levels; addressing gaps in consideration of patients and equity, diversity, and inclusion; and ensuring sufficient resourcing, including dedicated project management, clinician time, and evaluation capacity. Our findings suggest that AI may make important contributions in supporting service delivery; however, given the complex factors influencing procurement and deployment and the work involved in addressing these, AI may not address service challenges as straightforwardly as policymakers anticipate.

## Contributors

All authors contributed to the conceptualisation and development of the study.

Data curation: AIGR, NC, RL, HW, SB, ED, KH, EM, CSJ.

Accessed and verified underlying data: AIGR, NC, RL, HW.

Formal analysis: AIGR, NC, RL, HW.

Funding acquisition: AIGR, NC, HW, PLN, RM, SM, CSJ, NJF.

Investigation: AIGR, NC, RL, HW, SB, ED, KH, EM, CSJ.

Methodology: AIGR, NC, RL, HW, SB, ED, HE, FG, AH, KH, JL, EM, SM, RM, PLN, TOR, CSJ, NJF.

Project administration: HE, PLN.

Resources: AIGR, NC, RL, HW, SB, ED, HE, FG, AH, KH, JL, EM, SM, RM, PLN, TOR, CSJ, NJF.

Supervision: AIGR, HW, CSJ, SM, NJF.

Writing—original draft: AIGR.

TOR and FG provided clinical expertise and guidance.

All authors contributed to the interpretation of findings, visualisation, revising, and finalising the paper. All authors have read and approved the final manuscript.

## Data sharing statement

All data relevant to the study are included in the article or in the Supplementary information.

## Declaration of interests

AIGR is a trustee at Health Services Research UK. RM is Chair of the Board of Trustees of the Middlesex Association for the Blind; Vice-Chair on the Board of Trustees of the Research Institute for Disabled Consumers; Trustee on the Board of Thomas Pocklington Trust; Non-Executive Director on the Board of Evenbreak; and was Co-chair and Director on the Board of Shaping Our Lives to January 2025. SM is currently (2022-) a member of the Small Business Research Initiative (SBRI) Healthcare panel. His post is funded in part by RAND Europe, a non-profit research organisation. SM is also Deputy Director of Applied Research Collaboration East of England (NIHR ARC EoE) at Cambridgeshire and Peterborough NHS Foundation Trust. TOR is part of the AXREM AI Special Focus Group, the British Institute of Radiology AI Special Interest Group, NHS England AI Deployment Fund Oversight Committee and Society of Radiographers AI Advisory Group. FG is a shareholder in Optellum Ltd, is a co-founder and Chairman of the RAIQC Ltd, was an advisor to NICE on the use of chest x-ray AI in the NHS and a committee member of the RCR Advisory group. NJF is an NIHR Senior Investigator, a Non-Executive Director at Covid-19 Bereaved Families for Justice UK, a Non-Executive Director at Whittington Health NHS Trust until October 2024, a trustee at Health Services Research UK until 2022, a member of the UKRI and NIHR College of Experts for Covid-19 Research Funding (2020), the NIHR Health Services and Delivery Research (HS&DR) Programme Funding Committee (2013–2018), and the HS&DR Evidence Synthesis Sub Board (2016). All other authors report no conflicts of interest.
