# Supplementary material for: Procurement and early deployment of artificial intelligence tools for chest diagnostics in NHS services in England: a rapid, mixed method evaluation
Source: eClinicalMedicine. 2025 Sep 11;89:103481. doi: 10.1016/j.eclinm.2025.103481 (PMC12675025; doi:10.1016/j.eclinm.2025.103481)
Supplement: Supplementary files A–E [file mmc1.docx]

**Ramsay AIG et al ‘Procurement and early deployment of artificial intelligence tools for chest diagnostics in NHS services in England: A rapid, mixed method evaluation’**

[Supplementary file A. Interview topic guides 2](#_Toc205200467)

[Supplementary file A1. Network level interviews 2](#_Toc205200468)

[Supplementary file A2. Trust level interviews: Clinical staff: 5](#_Toc205200469)

[Supplementary file A3. Trust level interviews: PACS/IT/administrative staff: 8](#_Toc205200470)

[Supplementary File A4. AI supplier interviews 11](#_Toc205200471)

[Supplementary file B. RAP sheet 13](#_Toc205200472)

[Supplementary file C. Illustrative quotes from analysis 19](#_Toc205200473)

[Supplementary file D. Procurement panel membership 21](#_Toc205200474)

[Supplementary file E. Overview of AI suppliers who submitted tenders 22](#_Toc205200475)

**Note: list of abbreviations**

- AI=Artificial Intelligence
- AIDF=AI Diagnostic Fund (NHS England programme)
- CCIO= Chief Clinical Information Officer
- EDI=Equity, Diversity, Inclusion
- MASS= Managed Access Scheme (UK NHS program operated by NICE that permits access to promising but clinically uncertain new medicines while data are collected to inform long-term funding decisions)
- MHRA= Medicines and Healthcare products Regulatory Agency (UK regulator of medicines, medical devices and blood components for transfusion)
- NHS=National Health Service
- NICE=National Institute for Health and Care Excellence (health technology assessment body in England)
- IT= Information Technology
- PACS= Picture Archiving and Communication Systems
- QR Code= Quick Response code
- RAP Sheet= Rapid Assessment Procedure sheet – data collection tool

Supplementary file A. Interview topic guides

Supplementary file A1. Network level interviews

| **Topic** | **Main question/s** | **Follow-up questions/prompts** |
| --- | --- | --- |
| JOB ROLE | 1. Can you tell me about your current role? | - What are your main responsibilities? - Have you had any previous involvement with AI? - How have you been involved in the early deployment and implementation of AI for chest diagnostics? |
| UNDERSTANDING OF, AND ATTITUDES TOWARDS AI | 1. From your point of view, what do you think is driving the introduction of AI to support diagnostic reporting (and for chest specifically)? | - Agreement/disagreement with proposed use (whether they support it) - Anticipated impact - How AI can help and/or hinder the diagnostic process? - The role of AI to support diagnostics at their network specifically? - Why is AI needed? And why important for chest diagnostics for lung cancer? |
|  | 1. What are your thoughts/views about using AI to support chest diagnostics? | - Do you think implementation is needed/will be useful? |
| AI TOOL IN PRACTICE | 1. Could you describe the AI tool that you are currently using across your network? | - What is the purpose of the AI tool/proposed usage? - How is the use of the AI different from usual practice? |
|  | 1. Could you walk me through how the AI tool is used in practice? | How is AI used in practice as part of the wider clinical/patient pathway:   - Who receives initial referral? - Who uses the AI tool – who is the second reader? - How does the AI tool work? - What happens after the results are received? - Does the pathway differ across trusts? If so, how? |
| PROCESS OF PROCUREMENT  OBSTACLES AND ENABLERS  *(in context of procurement)* | 1. Can you walk me through the process of procurement at your network? | Key processes (where interviewee familiar):   - Proposal - Business case - Specification - Shortlisting - Assessment/specification - Contracting/selecting supplier   Broad questions:   - Which groups (and specific roles) were involved? - How did you consider EDI? - How did it work? - Did it change the pathway? If so, how? - How did scoping of evidence inform these processes? - Did these processes differ across trusts? If so, how? How was this variation managed? - What other factors influenced procurement? |
|  | 1. What helped your network during the procurement? | - Support from NHS – specific for AIDF - Regular meetings with trusts and/or within trusts - Communication – network to trust level but also being clear about what is required for networks and trusts - Staff capacity – at a network and trust level - Leadership and governance - Training/resources |
|  | 1. What made the process of procurement more challenging? | - Support or lack of – specific for AIDF - Communication (as above) - Leadership and governance - Training/resource - How have these obstacles been addressed or resolved? |
| PROCESS OF DEPLOYMENT AND ONGOING IMPLEMENTATION  OBSTACLES AND ENABLERS  *(in the context of early deployment and ongoing implementation)* | 1. Can you walk me through the process of early deployment and ongoing implementation at your network? | Key processes (where interviewee familiar **and involved**):   - Receiving funding - Terminology/programming for prioritisation - Shadowing phase (if used in their trust) - Start of using AI tool in practice   Broad questions:   - Which groups (and specific roles) are involved in implementation and usage? - How has it worked? - Has it changed the pathway? If so, how? - Experiences of collaboration |
|  | 1. How has the implementation of AI diagnostic tools progressed during the first few weeks/months? | - Uptake to date - Spread - Fidelity - Acceptance – staff, patients - Are the AI diagnostic tools being used in line with the aims and expectations you had as a network? - What other factors are influencing early implementation? Such as: Staff perceptions, engagement with and understanding of AI,   role of supplier, regulation |
|  | 1. What has helped your network during early deployment and ongoing implementation? | - Support from NHS – specific for AIDF - Regular meetings with trusts and/or within trusts - Communication – network to trust level but also being clear about what is required for networks and trusts - Staff capacity – at a network and trust level - Leadership and governance - Training/resources - Any unforeseen benefits |
|  | 1. What has been challenging for your network during early deployment and ongoing implementation? | - Staff perceptions, engagement with and understanding of AI - Role of supplier - Regulation - Support or lack of – specific for AIDF - Communication (as above) - Leadership and governance - Training/resource - Any unforeseen risks/ consequences - How have these obstacles been addressed or resolved? |
| IMPACT OF AI DIAGNOSTIC TOOLS | 1. What impact do you think the AI diagnostic tools are having and can continue to have moving forward? | Key areas to cover for clinical pathway:   - Potential impact on staff members (e.g., capacity, roles, satisfaction, stress) - Potential impact on patients (e.g., speed of referral, satisfaction) - Potential impact on a wider system level (e.g., the referral and diagnostic pathway for lung cancer) |
|  | 1. What impact do you think the AI tools may have for different population groups? | - Have services been doing anything to monitor the potential impact on inequalities? Or minimise any potential risk for certain groups? - If so, what have they done? How has this helped to address any risks? |
| MONITORING AND EVALUATION | 1. You have just mentioned what you think the impact is: do you have ways of assessing this impact across the network?   *[Preamble] We are interested in finding out about the data you collect to support the AI deployment because we want to know what is feasible for longer term evaluation.* | - (They might not be telling us anything specific about the data, yet, which is ok). - Depending on how they answer the first question we can ask the following questions. |
|  | 1. How is the data for the Benefits Register brought together? Can you walk us through the process of collecting data for the Benefits Register? | Are you collecting any additional information? For example:   - Related to the NICE guidelines? - To supplement local evaluation? - To evaluate impact on inequalities? - Patient experience? - Is there any key information that isn’t currently being captured in the Benefits Register that you would like to be? |
|  | 1. What, if any, are the challenges in collecting this data? | - Data quality and completeness - Too much data to collect - IT capabilities   *(to name a few examples)*   - How much time is required to engage with this for staff? Is there a dedicated staffing role for this? |
|  | 1. Are you able to monitor the accuracy of the AI tools? |  |
|  | 1. Are you aware if data collection processes vary across the network? |  |
| RESOURCES AND TRAINING | 1. What additional training (if any) have staff received to support the implementation of AI diagnostic tools across your network? | - What training resources have been used (e.g., workshops) - Have these been well received? - Any barriers/facilitators? - Is there any additional training needed? |
| REFLECTIONS AND THOUGHTS ON FUTURE PRACTICE | 1. What are key lessons you have taken away from procurement, deployment, and implementation? | - Anything unexpected? |
|  | 1. How do you think the process of implementation may continue to evolve moving forward? | - Future barriers/facilitators - Funding beyond 2 years - Do you think AI will continue to be used for diagnostics? - Would you recommend the use of AI in the future? |
| WRAP UP | 1. Is there anything else you would like to add that we haven’t discussed? |  |
| DEMOGRAPHICS | - Job role/title - Network/location - Length of time in role |  |

Supplementary file A2. Trust level interviews: Clinical staff:

| **Topic** | **Main question/s** | **Follow-up questions/prompts** |
| --- | --- | --- |
| JOB ROLE | 1. Can you tell me about your current role? | - What are your main responsibilities? - Have you had any previous involvement with AI? - How have you been involved in the early deployment and implementation of AI for chest diagnostics? |
| UNDERSTANDING OF, AND ATTITUDES TOWARDS AI | 1. From your point of view, what do you think is driving the introduction of AI to support diagnostic reporting (and for chest specifically)? | - Agreement/disagreement with proposed use (whether they support it) - Anticipated impact - How AI can help and/or hinder the diagnostic process? - The role of AI to support diagnostics at their trust specifically? - Why is AI needed? And why important for chest diagnostics for lung cancer? |
|  | 1. What are your thoughts/views about using AI to support chest diagnostics generally and in your trust specifically? | - Do you think implementation is needed/will be useful? |
| AI TOOL IN PRACTICE | 1. Could you describe the AI tool that you are currently delivering/using in your trust? | - What is the purpose of the AI tool/proposed usage? - How is the use of the AI different from usual practice? |
|  | 1. Could you walk me through how the AI tool is delivered /used in practice? | How is AI used in practice as part of the wider clinical/patient pathway:   - Who receives initial referral? - Who uses the AI tool – who is the second reader? - How does the AI tool work? - What happens after the results are received? - Does the pathway differ across trusts? If so, how? |
|  | 1. Can you describe the process of informing patients about the use of AI to support diagnostics at your trust? | - How have you ensured patient safety is maintained? - Have patients been told about the use of AI? If so, what information has been provided to them? If not, what implications do you think this may have? |
| PROCESS OF PROCUREMENT  OBSTACLES AND ENABLERS  *(in context of procurement)* | 1. Can you walk me through the process of procurement at your trust and your experiences of this? | - What were the incentives for implementation (if any)? - How did you consider EDI? - How were you involved as a trust? - What was your approach? - Which groups (and specific roles) were involved? - How did it work? (how did you go about making local arrangements/plans with the AI supplier?) - Did it change the pathway? If so, how? - How did scoping of evidence inform these processes? - How were you kept updated on the process? - What other factors influenced procurement? |
|  | 1. What helped your trust during the procurement? | - Support from NHS and network leads – specific for AIDF - Regular team meetings - Communication – network to trust level but also being clear about what is required for networks and trusts - Staff capacity - Leadership and governance - Training/resources |
|  | 1. What made the process of procurement more challenging? | - Support or lack of from NHS and network leads – specific for AIDF - Communication (as above) - Leadership and governance - Training/resource - How have these obstacles been addressed or resolved? |
| PROCESS OF DEPLOYMENT AND ONGOING IMPLEMENTATION  OBSTACLES AND ENABLERS  *(in the context of early deployment and ongoing implementation)* | 1. Can you walk me through the process of early deployment and ongoing implementation at your trust? | Key processes (where interviewee familiar **and involved**):   - Receiving funding - Terminology/programming for prioritisation - Shadowing phase (if used in their trust) - Start of using AI tool in practice   Broad questions:   - Which groups (and specific roles) are involved in implementation and usage? - How has it worked? - Has it changed the pathway? If so, how? - Experiences of collaboration |
|  | 1. How has the implementation of AI diagnostic tools progressed during the first few weeks/months? | - Uptake to date - Spread - Fidelity - Acceptance – staff, patients - Are the AI diagnostic tools being used in line with the aims and expectations you had as a trust and as a clinician? - Influence on care delivery (e.g., do clinicians still do the full review despite AI?) - What other factors are influencing early implementation? Such as: Staff perceptions, engagement with and understanding of AI,   role of supplier, regulation |
|  | 1. What has helped your trust during early deployment and ongoing implementation? | 1. Support from NHS and network leads – specific for AIDF 2. Regular team meetings 3. Communication – network to trust level but also being clear about what is required for networks and trusts 4. Staff capacity 5. Leadership and governance 6. Training/resources |
|  | 1. What has been challenging for your trust during early deployment and ongoing implementation? | - Staff perceptions, engagement with and understanding of AI - Role of supplier - Regulation - Support or lack of – specific for AIDF - Communication (as above) - Leadership and governance - Training/resource - Any unforeseen risks/ consequences - How have these obstacles been addressed or resolved? |
| IMPACT OF AI DIAGNOSTIC TOOLS | 1. What impact do you think the AI diagnostic tools are having and can continue to have moving forward? | Key areas to cover for clinical pathway:   - Potential impact on staff members (e.g., capacity, roles, satisfaction, stress) - Potential impact on patients (e.g., speed of referral, satisfaction) - Potential impact on a wider system level (e.g., the referral and diagnostic pathway for lung cancer) |
|  | 1. What impact do you think the AI tools may have for different population groups? | - Have services been doing anything to monitor the potential impact on inequalities? Or minimise any potential risk for certain groups? - If so, what have they done? How has this helped to address any risks? |
| MONITORING AND EVALUATION | 1. You have just mentioned what you think the impact is: do you have ways of assessing this impact across the trust?   *[Preamble] We are interested in finding out about the data you collect to support the AI deployment because we want to know what is feasible for longer term evaluation.* | - (They might not be telling us anything specific about the data, yet, which is ok). - Depending on how they answer the first question we can ask the following questions. |
|  | 1. How is the data for the Benefits Register brought together? Can you walk us through the process of collecting data for the Benefits Register? | Are you collecting any additional information? For example:   - Related to the NICE guidelines? - To supplement local evaluation? - To evaluate impact on inequalities? - Patient experience? - Is there any key information that isn’t currently being captured in the Benefits Register that you would like to be? |
|  | 1. What, if any, are the challenges in collecting this data? | - Data quality and completeness - Too much data to collect - IT capabilities   *(to name a few examples)*   - How much time is required to engage with this for staff? Is there a dedicated staffing role for this? |
|  | 1. Are you able to monitor the accuracy of the AI tools? |  |
|  | 1. Are you aware if data collection processes vary across the trust? |  |
| RESOURCES AND TRAINING | 1. What additional training (if any) have staff received to support the implementation of AI diagnostic tools at your trust? | - What training resources have been used (e.g., workshops) - Have these been well received? - Any barriers/facilitators? - Is there any additional training needed? |
| REFLECTIONS AND THOUGHTS ON FUTURE PRACTICE | 1. What are key lessons you have taken away from procurement, deployment, and implementation? | - Anything unexpected? |
|  | 1. How do you think the process of implementation may continue to evolve moving forward? | - Future barriers/facilitators - Funding beyond 2 years - Do you think AI will continue to be used for diagnostics? - Would you recommend the use of AI in the future? |
| WRAP UP | 1. Is there anything else you would like to add that we haven’t discussed? |  |
| DEMOGRAPHICS | - Job role/title - Network/location - Length of time in role |  |

Supplementary file A3. Trust level interviews: PACS/IT/administrative staff:

| **Topic** | **Main question/s** | **Follow-up questions/prompts** |
| --- | --- | --- |
| JOB ROLE | 1. Can you tell me about your current role? | - What are your main responsibilities? - Have you had any previous involvement with AI? - How have you been involved in the early deployment and implementation of AI for chest diagnostics? |
| UNDERSTANDING OF, AND ATTITUDES TOWARDS AI | 1. From your point of view, what do you think is driving the introduction of AI to support diagnostic reporting (and for chest specifically)? | - Agreement/disagreement with proposed use (whether they support it) - Anticipated impact - How AI can help and/or hinder the diagnostic process? - Why is AI needed? And why important for chest diagnostics for lung cancer? |
|  | 1. What are your thoughts/views about using AI to support chest diagnostics generally and in your trust specifically? | - Do you think implementation is needed/will be useful? |
| AI TOOL IN PRACTICE | 1. Could you describe the AI tool that you are currently delivering/using in your trust? | - What is the purpose of the AI tool/proposed usage? - How is the use of the AI different from usual practice? |
|  | 1. Could you walk me through how the radiology PACS/IT system is set up and works at your trust? | - Description of their usual IT system in radiology (also for lung cancer specifically) |
|  | 1. Could you walk me through how the AI tool has been introduced and integrated as part of the radiology PACS/IT system at your trust? | - Were you involved in any of the planning and preparation? If so, how were you involved? - Any challenges with this – if so, how were these challenges addressed? - Any facilitators/enablers? - Any unforeseen risks/ consequences? |
| PROCESS OF PROCUREMENT  OBSTACLES AND ENABLERS  *(in context of procurement)* | 1. Can you walk me through the process of procurement at your trust and your experiences of this? | - What were the incentives for implementation (if any)? - How did you consider EDI? - How were you involved as a PACS/IT staff member? If so, how did you approach this? - Did it change the pathway? If so, how? - How did scoping of evidence inform these processes? - How were you kept updated on the process? - What other factors influenced procurement? |
|  | 1. What helped your trust during the procurement? | - Support from NHS and network leads – specific for AIDF - Regular team meetings - Communication – network to trust level but also being clear about what is required for networks and trusts - Staff capacity - Leadership and governance - Training/resources |
|  | 1. What made the process of procurement more challenging? | - Support or lack of from NHS and network leads – specific for AIDF - Communication (as above) - Leadership and governance - Training/resource - How have these obstacles been addressed or resolved? |
| PROCESS OF DEPLOYMENT AND ONGOING IMPLEMENTATION  OBSTACLES AND ENABLERS  *(in the context of early deployment and ongoing implementation)* | 1. Can you walk me through the process of early deployment and ongoing implementation at your trust? | Key processes (where interviewee familiar **and involved**):   - Receiving funding - Terminology/programming for prioritisation - Shadowing phase (if used in their trust) - Start of using AI tool in practice   Broad questions:   - Which groups (and specific roles) are involved in implementation and usage? - How has it worked? - Has it changed the pathway? If so, how? - Experiences of collaboration |
|  | 1. How has the implementation of AI diagnostic tools progressed during the first few weeks/months? | - Uptake to date - Spread - Fidelity - Acceptance – staff, patients - Are the AI diagnostic tools being used in line with the aims and expectations you had as a trust and as a PACS/IT staff member? - What other factors are influencing early implementation? Such as: Staff perceptions, engagement with and understanding of AI,   role of supplier, regulation |
|  | 1. What has helped your trust during early deployment and ongoing implementation? | 1. Support from NHS and network leads – specific for AIDF 2. Regular team meetings 3. Communication – network to trust level but also being clear about what is required for networks and trusts 4. Staff capacity 5. Leadership and governance 6. Training/resources |
|  | 1. What has been challenging for your trust during early deployment and ongoing implementation? | - Staff perceptions, engagement with and understanding of AI - Role of supplier - Regulation - Support or lack of – specific for AIDF - Communication (as above) - Leadership and governance - Training/resource - Any unforeseen risks/ consequences - How have these obstacles been addressed or resolved? |
| IMPACT OF AI DIAGNOSTIC TOOLS | 1. What impact do you think the AI diagnostic tools are having and can continue to have moving forward? | Key areas to cover for clinical pathway:   - Potential impact on staff members (e.g., capacity, roles, satisfaction, stress) - Potential impact on patients (e.g., speed of referral, satisfaction) - Potential impact on a wider system level (e.g., the referral and diagnostic pathway for lung cancer) |
|  | 1. What impact do you think the AI tools may have for different population groups? | - Have services been doing anything to monitor the potential impact on inequalities? Or minimise any potential risk for certain groups? - If so, what have they done? How has this helped to address any risks? |
| MONITORING AND EVALUATION: Data collection metrics and processes | 1. What is your involvement in the Benefits Register data collection? | - Do you know how the data is brought together from other departments? - Do you link with data on patient comorbidities? - Do you know if the processes vary across the network? |
|  | 1. What is your level of involvement in collection of data in addition to the Benefits Register? | Are you collecting any additional information? For example:   - Related to the NICE guidelines? - To supplement local evaluation? - To evaluate impact on inequalities? - The point in which AI is used? - What kind of baseline data do you collect? Is it linked? - What’s the quality like? - Is there any key information that isn’t currently being captured in the Benefits Register that you would like to be?   *(Ask where appropriate)* |
|  | 1. Are you aware of any challenges in collecting this data? | - Data quality and completeness - Too much data to collect   *(to name a few examples)*   - How much time is required to engage with this for staff? Is there a dedicated staffing role for this? |
| MONITORING AND EVALUATION: IT capabilities | 1. What are your IT capabilities in data collection? | - Describe the infrastructure (e.g., data storage requirements, technical expertise, and resources you must collect for the Benefits Register and any other additional data?) |
|  | 1. What, if any, are the challenges in processing this data? | - Data governance issues - Impact of software updates etc. |
| RESOURCES AND TRAINING | 1. What additional training (if any) have staff received to support the implementation of AI diagnostic tools at your trust? | - What training resources have been used (e.g., workshops) - Have these been well received? - Any barriers/facilitators? - Is there any additional training needed? |
| REFLECTIONS AND THOUGHTS ON FUTURE PRACTICE | 1. What are key lessons you have taken away from procurement, deployment, and implementation? | - Anything unexpected? |
|  | 1. How do you think the process of implementation may continue to evolve moving forward? | - Future barriers/facilitators - Funding beyond 2 years - Do you think AI will continue to be used for diagnostics? - Would you recommend the use of AI in the future? |
| WRAP UP | 1. Is there anything else you would like to add that we haven’t discussed? |  |
| DEMOGRAPHICS | - Job role/title - Network/location - Length of time in role |  |

Supplementary File A4. AI supplier interviews

| **Topic** | **Main question/s** | **Follow-up questions/prompts** |
| --- | --- | --- |
| JOB ROLE, ORGANISATION AND PRODUCT INFORMATION | 1. Can you tell me about your organisation and current role? | - What are your main responsibilities? - How have you been involved in providing/ supplying AI for chest diagnostics? |
|  | 1. How long has your organisation been working with the healthcare sector and what have been your experiences of this? | - Have you had to make any adaptations |
|  | 1. Could you describe the type of AI products you currently supply? |  |
|  | 1. How is the AI tool intended to be used? | - How does it work? - How has it been tested/trained (EDI considerations)? - How is it being implemented into clinical pathways? |
| UNDERSTANDING OF, AND ATTITUDES TOWARDS AI | 1. From your point of view, what do you think is driving the introduction of AI to support diagnostic reporting in healthcare (and for chest specifically)? | - Agreement/disagreement with proposed use (whether they support it) - Anticipated impact - How AI can help and/or hinder the diagnostic process? - Why is AI needed? And why important for chest diagnostics for lung cancer? |
|  | 1. What are your thoughts/views about using AI to support chest diagnostics? | - Do you think implementation is needed/will be useful? |
| EXPERIENCE OF TENDERING/ COMMISIONING | 1. Could you walk me through the process of tendering/ commissioning your AI tool for networks? | - Specification - Assessment - Contracting - Competition |
|  | 1. What has helped you as an organisation during this process? | - Impact of these facilitators |
|  | 1. What has been challenging for you as an organisation during this process? | - Impact of these barriers - How were these addressed and overcome? |
| INVOLVEMENT WITH DEPLOYMENT AND ONGOING IMPLEMENTATION | 1. Can you walk me through the process of supplying AI tools for networks? | - Engaging with networks (e.g., meetings, demos) - Funding - Training (e.g., how much flexibility/tailoring for trusts and networks, considerations of EDI) - Rollout - Evaluation - Communication between leads/networks/suppliers and with MHRA/NICE/MASS |
|  | 1. How has the implementation of AI diagnostic tools progressed during the first few weeks/months? | - What has been your role in supporting ongoing implementation? - Any adaptations needed? |
|  | 1. What has helped you as an organisation when supplying AI tools to networks? | - Impact of these facilitators |
|  | 1. What has been challenging for you as an organisation when supplying AI tools to networks? | - Impact of these barriers - How were these addressed/overcome? |
|  | 1. How are your AI tools being implemented across different trusts (and networks if supplying to multiple networks)? | - What are the similarities and differences? - What is working well/not so well from your perspective? - Anything that could make things work better? (whether NHS or supplier) |
| IMPACT OF AI DIAGNOSTIC TOOLS | 1. As a supplier, what impact do you think the AI diagnostic tools are having and can continue to have moving forward? | Key areas to cover for clinical pathway (*may not all be relevant*):   - Potential impact on staff members (e.g., capacity, roles, satisfaction, stress) - Potential impact on patients (e.g., speed of referral, satisfaction) - Potential impact on a wider system level (e.g., the referral and diagnostic pathway for lung cancer) |
|  | 1. What impact do you think the AI tools may have for different population groups? | - What have they been doing as suppliers to address this and the wider challenges of EDI in AI? |
| MONITORING AND EVALUATION | 1. How do you assess or measure the impact of your AI tools? | - Do you provide any advice on how this may be achieved in practice (e.g., with NHS teams)? |
|  | 1. Are there any issues about different trusts using models of scanner which are different to the ones the algorithm was trained on? | - Why is that? - How have AI responded to issues (e.g., adaptive design?) |
|  | 1. Are there any issues concerning differences in the type of population the scanner was trained on to those on which it might be used in a hospital? | - Why is that? - How have AI responded to issues? |
| REFLECTIONS AND THOUGHTS ON FUTURE PRACTICE | 1. What are key lessons you have taken away from procurement, deployment, and implementation? | - Anything unexpected? |
|  | 1. How do you think the process of implementation may continue to evolve moving forward? | - Future barriers/facilitators - Funding beyond 2 years - Do you think AI will continue to be used for diagnostics? - Would you recommend the use of AI in the future? |
|  | 1. Was your tool used in any of the pilot studies with networks? | - **If yes:** Are you able to share any information from that study with us? |
| WRAP UP | 1. Is there anything else you would like to add that we haven’t discussed? |  |
| DEMOGRAPHICS | - Job role/title - Length of time in role - Supplier |  |

Supplementary file B. RAP sheet

| **Artificial Intelligence for Chest Diagnostics**  **RAP sheet** | | |
| --- | --- | --- |
| **Network: XXXX**  **Name of trusts:**   1. **XXXX** 2. **XXXX** | | |
| **Lead researcher:** | | |
| **Type of site:** In-depth | | |
| **Data included in RAP sheet:** *[number of interviews, number of documents etc- network and trust level]* | | |
| **Topic** | | **Main findings**  **Note: please add summary bullet points of notes from data collection activities. For each finding, please indicate the type of data that it came from (e.g. supplier, network, staff interview data, documents, meeting observation), and in brackets the interview ID number(s) the data can be attributed to* |
| **Site context** | Site context *(e.g. geographical area covered, which organisations involved in network, which organisations involved in evaluation, prior experience with AI tools for diagnostics)* | **Network level** |
|  |  | Trust 1 |
|  |  | Trust 2 |
| **About the AI tool** | Reason for change | **Network level** |
|  | Purpose of AI tool for network *(aims and goals)* | **Network level** |
|  |  | Trust 1 |
|  |  | Trust 2 |
|  | Description of AI tool | **Network level** |
|  | How AI tool will be used within the network (proposed usage, including variations across trusts) | **Network level** |
|  |  | Trust 1 |
|  |  | Trust 2 |
| **AI supported pathway – how AI is used/will be used** | Use of the AI tool for additional services:   - Is the AI tool being used in the delivery of additional service(s)? - If so, which? - Is this the primary use of the AI tool?   What are the key outcomes for these service(s)? | **Network level** |
|  |  | Trust 1 |
|  |  | Trust 2 |
|  | How radiology/IT system is set up | **Network level** |
|  |  | Trust 1 |
|  |  | Trust 2 |
|  | Referral process *(E.g. who receives initial referral)* | **Network level** |
|  |  | Trust 1 |
|  |  | Trust 2 |
|  | Who uses the tool *(e.g. who is the second reader?)* | **Network level** |
|  |  | Trust 1 |
|  |  | Trust 2 |
|  | What uses does the tool have? (lung cancer diagnosis, other (please list all) | **Network level** |
|  |  | Trust 1 |
|  |  | Trust 2 |
|  | How does the AI tool work? *(including prioritisation processes and what happens to cases not prioritised)* | **Network level** |
|  |  | Trust 1 |
|  |  | Trust 2 |
|  | What happens after the results are received? | **Network level** |
|  |  | Trust 1 |
|  |  | Trust 2 |
|  | How AI tool differs from current practice | **Network level** |
|  |  | Trust 1 |
|  |  | Trust 2 |
| **Service set up - procurement** | Process of procurement of AI tool at network level (*proposal, business case, specification, shortlisting, assessment/specification, contracting/selecting supplier) (e.g. which groups involved, considerations of EDI, changes to pathway, variation across trusts)* | **Network level** |
|  | Factors influencing procurement *(Barriers and facilitators)* | **Network level** |
| **Service set up – preparation for deployment** | Preparation for deployment *(including receiving funding, terminology/programming, working with supplier, who involved, how it’s worked, pathway changes)* | **Network level** |
|  |  | Trust 1 |
|  |  | Trust 2 |
|  | Factors influencing preparation for deployment *(Barriers and facilitators)* | **Network level** |
|  |  | Trust 1 |
|  |  | Trust 2 |
|  | Implementation stages of AI tool across network *(e.g. implemented all at once or in stages across trusts?)* | **Network level** |
| **Service implementation (including deployment)** | Date of implementation | **Network level** |
|  |  | Trust 1 |
|  |  | Trust 2 |
|  | Description of implementation to date | **Network level** |
|  |  | Trust 1 |
|  |  | Trust 2 |
|  | Communication/transparency of tool with patients | **Network level** |
|  |  | Trust 1 |
|  |  | Trust 2 |
|  | Experience of implementation *(including how much it is being used, how widely it is being used within trust, whether it is being used as intended within the aims and expectations)* | **Network level** |
|  |  | Trust 1 |
|  |  | Trust 2 |
|  | Factors influencing implementation (*Barriers and facilitators)* | **Network level** |
|  |  | Trust 1 |
|  |  | Trust 2 |
| **Perceived impact of AI diagnostic tools** | Perceived impacts on staff *(including inequalities, capacity, satisfaction etc)* | **Network level** |
|  |  | Trust 1 |
|  |  | Trust 2 |
|  | Perceived impacts on patients *(including inequalities, speed of referral, satisfaction)* | **Network level** |
|  |  | Trust 1 |
|  |  | Trust 2 |
|  | Perceived impacts for trust | **Network level** |
|  |  | Trust 1 |
|  |  | Trust 2 |
|  | Perceived impacts for network and/or wider context | **Network level** |
|  |  | Trust 1 |
|  |  | Trust 2 |
| **Staff experience** | Staff perceptions of and attitudes towards AI tools | **Network level** |
|  |  | Trust 1 |
|  |  | Trust 2 |
|  | Staff experiences of delivering healthcare using the AI tool | **Network level** |
|  |  | Trust 1 |
|  |  | Trust 2 |
|  | Staff training - description of training received | **Network level** |
|  |  | Trust 1 |
|  |  | Trust 2 |
|  | Views on training (e.g. things that work well, don’t work well, recommendations to improve) | **Network level** |
|  |  | Trust 1 |
|  |  | Trust 2 |
| **Future of the service** | Lessons for improvement | **Network level** |
|  |  | Trust 1 |
|  |  | Trust 2 |
|  | Sustainability and future implementation of these tools or similar tools | **Network level** |
|  |  | Trust 1 |
|  |  | Trust 2 |
| **Data, monitoring and evaluation** | Description of data collected *(including variations across and within network) for i) outcomes, i.e. patient related data, ii) process monitoring, i.e. system related data and iii) benefits realisation data* | **Network level:** |
|  |  | Trust 1 |
|  |  | Trust 2 |
|  | Process of collecting data *(including variations across and within network, frequency and continuity of monitoring, challenges)* | **Network level:** |
|  |  | Trust 1 |
|  |  | Trust 2 |
|  | Gaps in data collection | **Network level:** |
|  |  | Trust 1 |
|  |  | Trust 2 |
|  | Factors influencing data collection/capacity | **Network level** |
|  |  | Trust 1 |
|  |  | Trust 2 |
|  | Plans for local evaluation and whose role this is | **Network level:** |
|  |  | Trust 1 |
|  |  | Trust 2 |
| **Resources/costs** | Costs of AI tool *(including how the costs are funded, whether funds other than AIDF are being used, any additional resources coming through supplier)* | **Network level** |
|  |  | Trust 1 |
|  |  | Trust 2 |
|  | Staff resources/costs (*including how the costs are funded, whether funds other than AIDF are being used, any additional resources coming through supplier e.g. project management etc)* | **Network level** |
|  |  | Trust 1 |
|  |  | Trust 2 |
|  | Other non staff costs/resources (*including how the costs are funded, whether funds other than AIDF are being used, any additional resources coming through supplier e.g. training, etc)* | **Network level** |
|  |  | Trust 1 |
|  |  | Trust 2 |

Supplementary file C. Illustrative quotes from analysis

| Theme | Quote |
| --- | --- |
| ***1. Policy/service context*** | "It's being able to make the time to do it on top of still doing your other work as well. So that's the challenging this is: you don't give up your day job to be doing this extra stuff. You find the time to do the extra stuff" (consultant radiologist, Network 3) |
| ***2. Procurement*** |  |
| 2a. Convening panels | “I think we made a good team, because there was the clinical input, alongside the technical procurement things. So I was learning off them, and they were learning off me” (Consultant radiologist, Network 5) |
| 2b. Assessing and selecting tenders | “we’re wanting to buy a car but we’re choosing that car based on just reading the brochure and not going on a test drive for it. And I think a lot of colleagues were very keen on having a session where the various softwares were setup on workstations and we could go to the workstation, use the workstation and evaluate the software that way." (consultant radiologist, Network 6)  “some of the clinicians, especially the specialists had a little bit of bias in their head, they can’t help it. ‘I worked with so and so before and I know how it works so therefore that’s the one I want’.” (Innovation lead, Network 10)  “unfortunately those questions, they tend to get less attention even like the social value ones from stakeholder groups. They just see them as a bit of a box-ticking exercise and maybe like the carbon reduction and net zero” (procurement specialist, Network 9)  “we definitely discussed the different cohorts of populations, even across the sector because our patients are very different, even though we’re quite geographically close, so that came up […] We definitely reviewed their training data set and the make-up of that.” (Programme manager, Network 9)  “There was a lot of information provided by the suppliers. Too much. Far too much and far too much technical detail to go through. That’s possibly a negative thing, on their part, to expect clinicians to go through a lot of details that they'd provided. It was overwhelming the amount of information that was provided to us. There was a lot of links to those documents throughout some of the responses as well.  Again, that was an area that helped to differentiate the score, but possibly not for the best reasons because we were possibly missing really good, valuable information but it was hidden within vast numbers of documents.” (Consultant radiologist, Network 1) |
| 2c. Contracting | “They were challenging our judgement overall and saying that they were the best tender and they should be awarded the contract overall and being very obstructive to the overall process” (Programme manager, Network 2)  “chasing with both third parties and the Trusts to get them signed off by the right people, all gone through internal processes and things like that. So that ate into it quite a bit because they can't start working with the Trusts until that’s been paid, so we had to obviously get that through, receipted and paid. That was a lengthy process because of the amount of Trusts involved” (project manager, Network 1) |
| ***3. Preparation for deployment*** |  |
| 3a. integrating AI with local IT systems | “It has just been an incredible amount of testing which I have been involved with some of it to make sure that it is working correctly on the correct patients and the systems overall are all integrated” (Radiographer, Network 1) |
| 3b. obtaining local governance permissions | “Ideally, a consultant who could be in charge at the local hospital of this software – so how they want to implement it in their hospital. They are responsible for getting it through any of their own local governance processes. And they are responsible for speaking with their thoracic surgeons and getting that up and running. So, that was something that we stipulated” (Programme manager, Network 2)  “[Trust] have got an actual project manager on the project, which is why they're flying ahead, and it’s been really well coordinated. The other Trusts have put like the PACS administrator or the radiology manager […] as their project lead and they're not really project leads as such. So, it’s been difficult with the other Trusts just to keep everything moving along.” (Network manager, Network 1)  “think the real challenge we’ve had is the clinical safety report required. I would probably say I would never underestimate the length of time this might actually end up taking […] our challenge is getting things in a timely fashion that allow us to have those conversations […] to sign that off in their organisation because we don’t have the support internally” (Network lead, Network 8)  “I’m sure there are documents already that they've done but it doesn't translate to the Trust. The Trusts don't accept you know that, they want to have their own kind of peace of mind around the information governance and understanding you know, if there's any safety issues” (PACS manager, Network 6)  "We have always had a bit of a bottleneck with the IG stuff because you fill in a form and it takes them, they have got a turnaround time which is a few weeks sometimes. And you are back and forth, there are queries.” (Trust project manager, Network 1) |
| 3c. engaging and training staff | “it's not a big change apart from just knowing, looking at the findings […] provided by the AI but radiographers, those who perform the X-rays are going to be trained. There is a training video that we're going to enrol, we don't want to enrol it now because they might, they might forget how to do it, they want to enrol it when it's closer to time” (Radiographer, Network 3)  “With all of these things, it's letting people know things are happening and things are going on to give people an awareness of what's going on in the background but then there's so much else that goes on, if you tell people too much too quickly then you tend to find that it gets forgotten about or you sit and think well that's the job done. Job tick, done. And then you actually have to go back and repeat it or repeat it again and again and again and again. So it's a matter of trying to do it in a timely fashion so that you're informing people when they need to know.” (Consultant radiologist, Network 3)  “A lot of senior colleagues who’ve been around for ages, they… All of them don’t think it’s a good idea. They think the same […] that the quality and the standards, that’s probably going to drop because you’ve got a computer literally telling you that, you know- This is the message that’s going to come out of it. But the younger kind of registrars, people, new consultants coming in, they’re more with it. […] It doesn’t matter how much you tell them it’s going to help you and it’s going to do this for you and it’s going to speed your time up, but a lot of people are thinking, you know, ‘am I really worried about speed when it comes to looking at something clinically and getting that right?’ So, you wouldn’t stand up in court and say, you know, they asked it to help me and I was trying to report very quickly to get my backlog done” (PACS manager, Network 9) |
| 3d. considering patient engagement and equity, diversity, and inclusion | “the intended use of the AI, we’re piloting it, it’s not replacing it’s providing a system essentially just to move patients around on the worklist and so a human being can look at those sooner rather than later. Our CCIO, <name 6>, having understood that said he didn’t feel that it’s something we needed to tell patients, but as a bare minimum we should probably put things like posters up in waiting rooms with QR codes so that if a patient is interested they can scan and get a bit more information about what we’re doing and how it works” (Network lead, Network 8) |
| 3e. providing data to evaluate impact | “It's actually quite a complicated data request I think that's come out from the national team and organisations don't typically have analysts in place to do this work permanently” (consultant radiologist, Network 4)  “We’ve really struggled with <organisation> locally to get the right data, some of which was just missing or the system didn’t allow them to track the right data or parameters” (Network lead, Network 8)  “The reason is that [network] have been quite lucky as you know we’re a mature network, been around for over 10 years. We’re already running a BI tool and what we’ve set up is a new set of dashboards that monitor the AI or where it’s going to be applied. So we can benchmark existing turnaround terms of stats and then review that as it goes forward and do that as and when we want to” (Innovations lead, Network 10) |

Supplementary file D. Procurement panel membership

| **Network** | **Procurement panel membership** |
| --- | --- |
| 1 | Panel of 9, including: Radiologists; Service manager; Radiographer; Systems experts; Imaging network team, including procurement experts |
| 2 | Panel of 9, including: radiologist; Medical Director; Head of Imaging operations; Lead accountant; Imaging network team |
| 3 | Panel of 15, including: Radiologists; Radiographers; PACS; Digital imaging lead; Radiology system lead; Chest specialists; Data analyst; Project manager; Imaging network team |
| 4 | Not available. |
| 5 | Panel of 6, including: Radiologist/AI lead; Clinical AI fellow; Cross-site Radiology manager; IT enterprise architect; Head of IG; Cyber security manager |
| 6 | Panel of 13, including: Radiologists; PACS; Imaging Manager; Chief Technology Officer; Director of Finance; Senior Accountant; Imaging network team |
| 7 | Not available. |
| 8 | Panel of 10, including: Radiologists, Radiographers, Digital transformation lead, PACS, Chief Medical Officer, General Manager, ICB, Imaging network team |
| 9 | Panel of 14, including: Radiologists; Regional Data Protection Officer; IT managers; Imaging manager; Independent consultant; ICB (digital transformation); Imaging network team |
| 10 | Not available. Interviews confirm panel included representatives across the different member trusts. |

Supplementary file E. Overview of AI suppliers who submitted tenders

|  | Network | | | | | | | | | |
| --- | --- | --- | --- | --- | --- | --- | --- | --- | --- | --- |
| Supplier | **1** | **2** | **3** | **4^a^** | **5** | **6** | **7^a^** | **8** | **9** | **10** |
| 1 |  |  |  |  |  | Y |  |  |  |  |
| 2 |  | Y |  |  |  | Y |  |  |  | Y |
| 3 | Y | Y | Y |  | Y |  |  |  | Y | Y |
| 4 | Y | Y |  |  | Y |  |  |  | Y | Y |
| 5 |  | Y |  |  |  |  |  |  | Y |  |
| 6 |  | Y |  |  | Y | Y |  | Y |  |  |
| 7 |  |  |  |  |  | Y |  |  |  |  |
| 8 | Y | Y | Y |  |  | Y |  |  | Y | Y |
| 9 | Y | Y |  |  | Y |  |  | Y | Y | Y |
| 10 |  |  |  |  |  |  |  |  | Y |  |
| 11 | Y | Y | Y |  | Y | Y |  | Y | Y | Y |
| 12 |  |  |  |  | Y |  |  | Y | Y |  |
| 13 |  |  | Y |  |  |  |  |  |  |  |
| 14 |  |  | Y |  |  |  |  |  |  |  |
| 15 |  |  | Y |  |  |  |  |  |  |  |
| 16 |  | Y |  |  |  | Y |  |  | Y | Y |

Note. ^a^=data not available; Y=‘tender submitted’
